# Favourable IFNL3 Genotypes Are Associated with Spontaneous Clearance and Are Differentially Distributed in Aboriginals in Canadian HIV-Hepatitis C Co-Infected Individuals

**DOI:** 10.3390/ijms16036496

**Published:** 2015-03-20

**Authors:** Nasheed Moqueet, Claire Infante-Rivard, Robert W. Platt, Jim Young, Curtis Cooper, Mark Hull, Sharon Walmsley, Marina B. Klein, the Canadian Co-Infection Study Investigators

**Affiliations:** 1 Department of Epidemiology, Biostatistics and Occupational Health, McGill University, Montreal, QC H3A 1A2, Canada; E-Mails: nasheed.moqueet@mail.mcgill.ca (N.M.); claire.infante-rivard@mcgill.ca (C.I.-R.); robert.platt@mcgill.ca (R.W.P.); 2 Basel Institute for Clinical Epidemiology and Biostatistics, University Hospital Basel, Basel 4031, Switzerland; E-Mail: jyoung@uhbs.ch; 3 Department of Medicine, Division of Infectious Diseases/Chronic Viral Illness Service, Royal Victoria Hospital, McGill University Health Centre, 3650 Saint-Urbain Street, Montreal, QC H2X 2P4, Canada; 4 The Ottawa Hospital—Research Institute, Ottawa, ON K1Y 4E9, Canada; E-Mail: ccooper@toh.on.ca; 5 BC Centre for Excellence in HIV/AIDS, Vancouver, BC V6Z 1Y6, Canada; E-Mail: mhull@cfenet.ubc.ca; 6 Toronto General Research Institute, University Health Network, University of Toronto, Toronto, ON M5G 2M9, Canada; E-Mail: sharon.walmsley@uhn.ca; 7 The Canadian Co-Infection Study Investigators (see Appendix)

**Keywords:** Aboriginals, genetic factors, Hepatitis C spontaneous clearance, Hepatitis C epidemiology, HIV-hepatitis C co-infection, interferon lambda 3

## Abstract

Canadian Aboriginals are reported to clear Hepatitis C (HCV) more frequently. We tested the association of spontaneous clearance and three single nucleotide polymorphisms (SNPs) near the *Interferon-lambda 3 (IFNL3)* gene (rs12979860, rs8099917, functional variant rs8103142) and compared the SNP frequencies between HIV-HCV co-infected whites and Aboriginals from the Canadian Co-infection Cohort. HCV treatment-naïve individuals with at least two HCV RNA tests were included (*n* = 538). A spontaneous clearance case was defined as someone with two consecutive HCV RNA-negative tests, at least six months apart. Data were analyzed using Cox proportional hazards adjusted for sex and ethnicity. Advantageous variants and haplotypes were more common in Aboriginals than Caucasians: 57% *vs.* 46% had the rs12979860 CC genotype, respectively; 58% *vs.* 48%, rs8103142 TT; 74% *vs.* 67%, the rs12979860 C allele; and 67% *vs.* 64% the TCT haplotype with three favourable alleles. The adjusted Hazard Ratios (95% CI) for spontaneous clearance were: rs12979860: 3.80 (2.20, 6.54); rs8099917: 5.14 (2.46, 10.72); and rs8103142: 4.36 (2.49, 7.62). Even after adjusting for rs12979860, Aboriginals and females cleared HCV more often, HR (95% CI) = 1.53 (0.89, 2.61) and 1.42 (0.79, 2.53), respectively. Our results suggest that favourable IFNL3 genotypes are more common among Aboriginals than Caucasians, and may partly explain the higher HCV clearance rates seen among Aboriginals.

## 1. Introduction

Among those infected with Hepatitis C (HCV), approximately 20%–45% spontaneously clear the infection without treatment [1]. This proportion is lower in HIV co-infected individuals due to weaker HCV-specific immune responses. Factors associated with higher HCV clearance include female sex, East Asian ancestry, and infection with HCV genotypes 2 and 3 [2,3,4,5,6,7,8]. In Canada, Aboriginal ancestry has also been associated with spontaneous clearance [9,10]. HIV coinfection, on the other hand, is associated with lower rates of spontaneous clearance [11]. Of the host genetic factors linked with favourable HCV outcomes, the most consistent have been the single nucleotide polymorphisms (SNPs) in the *Interferon-lambda 3* (*IFNL3*) gene, formerly *IL28B* [12,13,14,15].

The SNPs near the *IFNL3* gene (rs12979860 and rs8099917) are strongly predictive of spontaneous clearance of HCV and favourable treatment response, in both mono [1,2,3,16,17,18,19,20,21,22] and co-infected [4,11] populations. The odds of spontaneous clearance are three times higher in those inheriting two copies of the beneficial alleles *versus* those with one or no copies. Other SNPs that have been proposed as the causal mechanism include ss469415590 (*IFNL4*) which impairs HCV clearance by turning on hepatic interferon-stimulated genes (ISGs) and reducing responsiveness to IFNL3 [23,24,25] or rs8103142, which leads to amino acid substitutions in the IFNL3 protein [26,27]. The SNPs also continue to be a strong determinant of treatment response with the more efficacious direct acting antiviral agents (DAAs) [28]. For example, SNP rs12979860 can be used to identify candidates for shorter treatment duration [29] and IFNL3 SNPs are predictive of SVR with interferon-free regimens [30,31].

Allele frequencies of the three IFNL3 SNPs vary among ethnicities, with the favourable variants being almost universal in East Asians and rare in those of African ancestry [1,16,18,19]. However, no studies on *IFNL3* have been conducted in HCV-infected Canadian populations, which are unique in their genetic makeup. Aboriginals accounted for 15.2% of new HCV infections between 1999–2004 [32,33], despite representing only 4.3% of the Canadian population [34]. In the HIV-HCV co-infected population in the Canadian Co-infection Cohort (CCC), 16% of participants report Aboriginal descent. Despite higher HCV seroprevalence in mono-infected Aboriginals [35], less than 5% had detectable HCV RNA compared with 75% of non-Aboriginal Canadians in one study [36]. This finding suggests Aboriginals may have markedly increased rates of HCV clearance possibly due to specific immunological responses [10,14,15,36,37,38]. Aboriginals also respond better to HCV treatment in some studies [33,39] but not all [40]. The distribution of IFNL3 SNPs in Aboriginals, which could differ from Caucasians due to their separate historical and genetic ties to populations in central Asia as well as their distinct migration and mixing patterns [41,42], has never been reported. Given the burden of infection in the Aboriginal population, information on IFNL3 would be valuable for tailoring treatment for co-infected Aboriginals who face numerous challenges in accessing care.

We studied the association between IFNL3 SNPs (rs12979860, rs8099917 and rs8103142) and spontaneous clearance and compared the distribution of IFNL3 SNPs in Canadian whites and Aboriginals.

## 2. Results

For the spontaneous clearance study, the study population included 46% of those enrolled in the CCC. The distribution of the sociodemographic and clinical features of included patients were similar to the CCC as a whole (Table 1). Half the participants had been infected with HCV for over 18 years. About 90% were HCV RNA positive at their first available test and very few were ever treated for HCV. Twenty-two of the 79 (28%) spontaneous clearance cases were HCV RNA positive at cohort entry and cleared HCV infection while under observation. The majority were male, older, with a history of injection drug use and were therefore likely infected with HCV before acquiring HIV. The majority were receiving antiretroviral therapy and had well-controlled HIV with good CD4 recovery (median CD4 count at baseline >350 cells·μL^−1^). The 85 Aboriginals in the study population for the IFNL3-spontaneous clearance study were more likely to be female (60% *vs.* 32%) and their median CD4 count was slightly lower at 330 cells·μL^−1^ (*vs*. 365 cells·μL^−1^ in the study population). Spontaneous clearance cases were more likely to be female, Aboriginal, less likely to be infected with genotype 1, and more likely to possess the advantageous genotypes at all the IFNL3 SNPs than those who did not clear spontaneously (Table 2).

### 2.1. Clearance and IFNL3

Eighty individuals (15%) cleared spontaneously; one was missing HCV duration information, so 79 cases were used in the analysis. IFNL3 alleles were in Hardy-Weinberg equilibrium in both whites and Aboriginals (*p* > 0.01). The favourable genotypes at all the SNPs were associated with higher rates of clearance at a statistically significant level, with hazard ratios >3 in both univariate and multivariate analyses (Table 2 and Table 3). The rates of clearance did not change appreciably after adjustment for ethnicity or sex, indicating that the effect of the SNP is likely not related to, or mediated by, either of these variables.

**Table 1:** Baseline characteristics of IFNL3-spontaneous clearance study population compared with the Canadian Co-infection Cohort (CCC) source population.

Variables	Study Population *n* = 538	CCC *n* = 1176
Median follow-up time, years (IQR)	3.2 (1.7–4.6)	3.0 (1–4.4)
Mean age at baseline, years (SD)	44 (8.2)	45 (8.6)
Male, *n* (%)	368 (68)	864 (74)
Ethnicity, *n* (%)		
White	418 (78)	891 (77)
Black	15 (3)	45 (4)
Aboriginal	85 (16)	181 (16)
Other	15 (3)	44 (4)
Injection drug use ever, *n* (%)	472 (87)	944 (81)
Median HCV duration, years (IQR)	19 (11–25)	18 (10–26)
HCV RNA positive at first available test	481 (90)	889 (76)
HCV genotype 1, *n* (%)	304 (74) ^a^	683 (73) ^b^
Median CD4 counts, cells/μL (IQR)	365 (230–530)	420 (270–604)
On HIV therapy	415 (77)	957 (81)

^a^ HCV genotype available in 410 individuals at visit 1; ^b^ HCV genotype available in 935 individuals at visit 1.

**Table 2:** Characteristics of spontaneous clearers compared to chronically HCV infected patients and univariate Cox proportional hazards analyses of spontaneous HCV clearance.

Variables	Spontaneous Clearers *n* = 79	Chronically Infected *n* = 462	Univariate HR (95% CI)
Aboriginal	18 (23%)	67 (15%)	1.91 (1.12, 3.25) ^a^
Female	32 (41%)	142 (31%)	1.62 (1.02, 2.57)
HCV genotype, *n* (%)			
1	11 (52%)	293 (75%)	0.56 (0.29, 1.10) ^b^
2	1 (5%)	17 (4%)	
3	9 (43%)	67 (17%)	
4	0	12 (3%)	
	IFNL3 genotypes		
rs12979860			
CC, *n* (%)	53 (75%)	180 (43%)	3.89 (2.28, 6.63) ^c^
CT, *n* (%)	15 (21%)	186 (44%)	
TT, *n* (%)	3 (4%)	57 (13%)	
rs8099917			
TT, *n* (%)	68 (88%)	256 (60%)	4.65 (2.32, 9.32)
GT, *n* (%)	9 (12%)	138 (33%)	
GG, *n* (%)	0 (0)	30 (7%)	
rs8103142			
TT, *n* (%)	59 (78%)	186 (45%)	4.23 (2.46, 7.28)
CT, *n* (%)	15 (20%)	182 (44%)	
CC, *n* (%)	2 (2%)	50 (12%)	

^a^ The comparison shown is for Aboriginals *vs.* White Canadians (other ethnicities excluded); ^b^ HCV genotypes 1, 4 *vs.* genotypes 2, 3, after multiple imputation; ^c^ CC *vs.* non-CC.

**Table 3:** Multivariate results of the association of HCV spontaneous clearance with IFNL3 genotypes.

Characteristic	Adjusted HR (95% CI) by IFNL3 Genotype		
	rs12979860 CC	rs8099917 TT	rs8103142 TT
IFNL3 Genotype	3.80 (2.20, 6.54)	5.14 (2.46, 10.72)	4.36 (2.49,7.62)
Aboriginal Ethnicity *vs.* White	1.42 (0.79, 2.53)	1.50 (0.88, 2.57)	1.40 (0.79, 2.48)
Other Ethnicity *vs.* White	1.04 (0.21, 5.17)	1.19(0.25, 5.75)	1.01 (0.20, 5.05)
Female	1.53 (0.89, 2.61)	1.58 (0.97, 2.57)	1.55 (0.94, 2.56)

In both univariate and multivariate analyses, being of Aboriginal descent was linked to a higher likelihood of spontaneous HCV clearance (Table 2 and Table 3). The estimated clearance rates per 100 person-years (95% CI) for Aboriginals was 8.20 (5.17, 13.01) compared to 4.24 (3.28, 5.48) for whites, supporting the univariate estimate that Aboriginals were almost twice as likely to clear HCV compared with whites. There was no evidence that the IFNL3-spontaneous clearance relationship varied by ethnicity. Interaction terms between each SNP or haplotype with sex were not statistically significant (*p*-value = 0.9, results not shown). Females also appeared to have higher likelihood of clearing spontaneously, with a 50% higher clearance rate than males. Infection with HCV genotype 1 or 4 tended to be associated with a lower chance of clearance compared to infection with type 2 or 3 (HR = 0.56, 95% CI 0.29, 1.10 after multiple imputation for missing genotypes).

Patients carrying a haplotype with advantageous alleles from all three SNPs had a much greater rate of clearance than those lacking the haplotype, regardless of the mode of inheritance. A recessive model assumes that only having two copies of the beneficial haplotype raises the rate of clearance, while an additive model is closer to a dose response where two copies of the beneficial haplotype has a two-fold effect on the outcome compared to only one copy. Based on the AIC values, the additive model fit best, indicating that those with two copies of the haplotype were over three times more likely to clear than those with a single copy of the haplotype (OR = 3.23, 95% CI: 2.66, 3.92).

### 2.2. IFNL3 Distribution in Aboriginals and Whites

**Figure 1:**
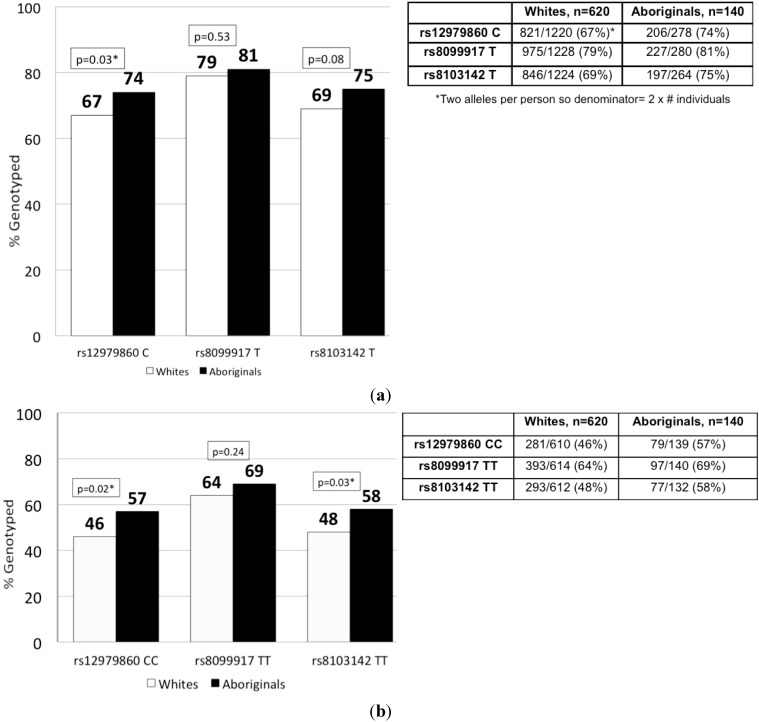
Distribution of favourable IFNL3 genotypes and alleles in Canadian-born Whites and Aboriginals: (**a**) Frequency of favourable IFNL3 alleles is higher in Aboriginals than Whites; (**b**) Frequency of favourable IFNL3 genotypes is higher in Aboriginals than Whites. * *p* < 0.05.

The prevalence of the beneficial alleles and the genotypes (Figure 1) was more common in Aboriginals than white Canadians, especially at rs8103142 and rs12979860 (*p* < 0.05). At rs8099917, the differences in the allelic and genotypic frequencies did not reach statistical significance (*p* = 0.53 and *p* = 0.24, respectively).

The SNPs were in linkage disequilibrium in both Aboriginals and whites (*p* < 0.001 in each), though the magnitude of the measures differed. Among whites, higher R^2^ and D’ values indicated the SNPs were much more tightly linked to rs12979860 than in Aboriginals. In whites, D’ values between favourable alleles in rs12979860 and rs8099917 were 0.85 (95% CI 0.79, 0.90) and between rs12979860 and rs8103142 were 0.93 (95% CI 0.89, 0.96). The corresponding values among Aboriginals were 0.68 (95% CI 0.53, 0.80) and 0.75 (95% CI 0.64, 0.83), respectively. In whites, R^2^ between rs12979860 and rs8099917 was 0.39 and between rs12979860 and rs8103142 was 0.81, while in Aboriginals, the values were 0.32 and 0.55, respectively.

**Figure 2:**
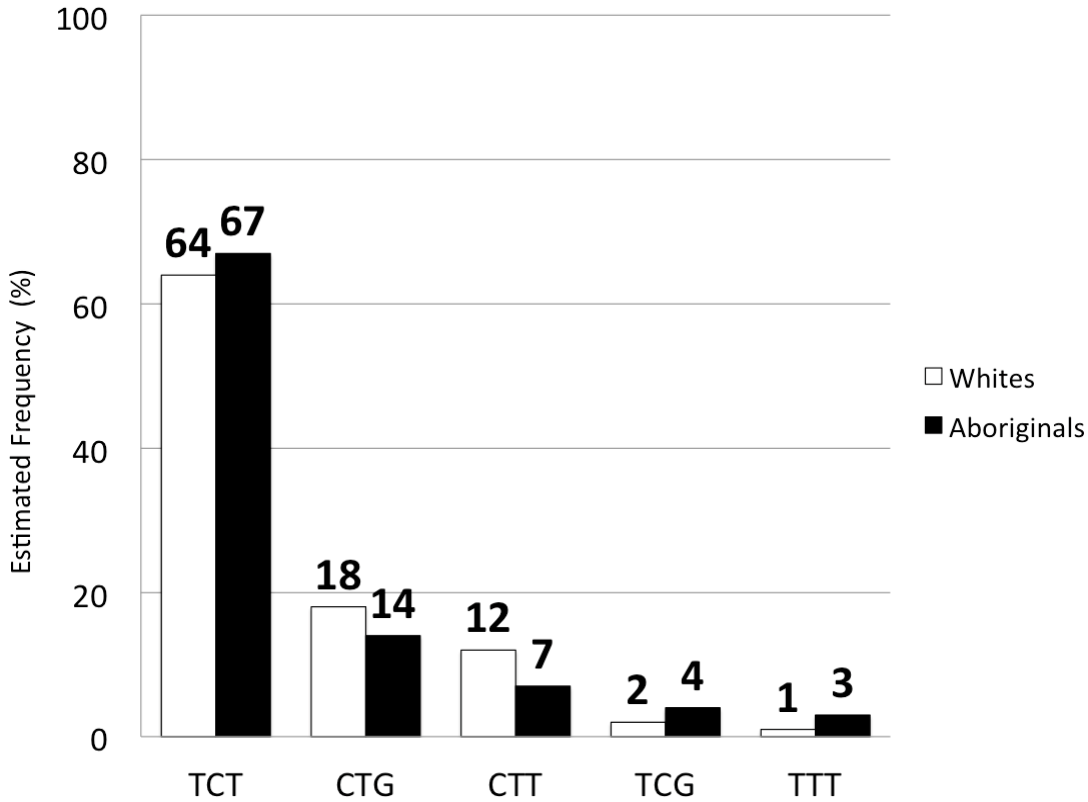
Haplotype distribution in Canadian-born Whites and Aboriginals. Haplotypes containing the favourable alleles at all three SNPs (TCT) are more common in Aboriginals than whites while the opposite is true about haplotypes with the disadvantageous alleles (CTG). TCT = T at rs8103142, C at rs12979860 and T at rs8099917.

Haplotype analysis with PHASE estimates the prevalence of the most common haplotypes in each population. The top five most common haplotypes are presented in Figure 2. Estimates indicate that the haplotype containing the beneficial allele at all three SNPs (TCT corresponds to T at rs8103142, C at s12979860 and T at rs8099917) was most frequent in both Aboriginals and whites, though more common in the former: 67% of Aboriginals *vs.* 64% of whites. On the other hand, CTG, which contains the unfavourable allele at each SNP, was more common in whites (18%) than Aboriginals (14%). Likelihood ratio test results also indicate that haplotypic frequency differed between the two groups (*p* < 0.05).

## 3. Discussion

Beneficial SNPs near the *IFNL3* gene, linked to both spontaneous HCV clearance and treatment response, are distributed differentially in populations of different ancestry, and have never been characterized in the Canadian HIV-HCV co-infected population where Aboriginal people are overrepresented. As in other studies, we found clearance rates in our coinfected cohort to be lower than in HCV mono-infected populations [4,43,44]. Aboriginals cleared HCV infection more often than whites and also possessed higher frequencies of the advantageous genotypes, alleles and haplotypes. Furthermore, after adjusting for beneficial IFNL3 genotypes, Aboriginals still tended to clear more often suggesting there may be additional factors that explain higher rates of spontaneous clearance in this population. Our results could have implications for treatment-related decisions, especially since Aboriginals are disproportionately affected by both HCV and HIV and more often carry favourable haptotypes that predict favourable HCV treatment responses.

SNPs of interest included those at rs12979860 and rs8099917, which are located in the non-coding region of the IFNL3 protein. We also studied the SNP at rs8103142, which leads to a nonsynonymous amino acid change (K70R), that could affect IFNL3 protein function [45,46] or interactions with other unknown factors involving viral control [47]. As in other populations, the favourable genotypes at all these SNPs were linked to higher rates of spontaneous clearance in HIV-HCV co-infected Canadians. The beneficial genotypes were more frequent in Aboriginals and the differences reached statistical significance at rs12979860 and rs8103142. Canadian Aboriginals, like other Native American indigenous peoples, have complex ancestries but share links with Asian populations (*i.e*., Siberians and Mongolians), where the beneficial genotypes are almost universal [15,41]. We found the IFNL3 allele frequencies in Canadian-born whites similar to those reported for European populations in other studies [48,49].

The frequency of IFNL3 haplotypes also differed between Aboriginals and Canadian-born whites. The TCT haplotype, which contains beneficial alleles at all three SNPs, was more common among Aboriginals than whites while the reverse was true for the CTG haplotype, which includes the unfavourable allele at each SNP. As with allelic frequencies, the haplotypic frequencies in whites were similar to those reported in other studies of Caucasian populations [23]. The frequencies of the beneficial alleles and haplotypes in Aboriginals were not as high as those reported in East Asians [15,23,41], suggesting genetic divergence or reflecting mixing with European or other populations over time—for example, 93% of East Asians carry the TCT haplotype *vs.* 67% of Canadian Aboriginals, and only 5.5% carry the unfavourable CTG version *vs.* 14% of Aboriginals [23].

As in prior studies [10,33,36,50], in univariate analyses, Aboriginals were more likely to clear than whites. This association weakened after adjustment for IFNL3 and sex. However, Aboriginals still appeared more likely to clear HCV than whites suggesting that IFNL3 may be only one factor that contributes to increased clearance among Aboriginals. Differences in killer-cell immunoglobulin-like receptors (KIR) or IL-10 variants may explain higher HCV resolution among Aboriginals [14,15]. We could not examine these factors, or test if they interact with the SNPs near *IFNL3*.

The effect of rs8103142 cannot be separated from rs12979860 at a population level, making it difficult to conclude if the SNPs were directly linked to the biological mechanism of spontaneous clearance or rather were behaving as markers for the true causal variant. The lysine-arginine (K70R) substitution caused by the rs8103142 polymorphism did not affect IFNL3 protein function in *in vitro* studies [51,52], but since these studies involved a single experimental model within a short time frame (24 h), the authors could not rule out a major role for the rs8103142 variant in treatment response [52,53]. It is also possible that rs8103142 alleles are in LD with other causal variants such as ss469415590, which encodes Interferon Lambda 4 (IFNL4) [54]. Some linkage (R^2^ ≥ 0.6) between the IFNL4 SNP and both rs12979860 and rs8103142 have been reported in other studies [23,24].

The strengths of our study include a study sample that is representative of the co-infected population in Canada and included a large number of Aboriginal persons, so our findings will be directly relevant to treating clinicians. Our cases were carefully defined and sampled to reduce measurement error, thus providing reasonable estimates of relative rates of clearance.

Although ours is the largest study to date of spontaneous clearance in co-infected Canadians, we could not study other host or viral factors that could impact the IFNL3-spontaneous clearance relationship. As expected, infection with HCV genotype 1 or 4 was linked to a much lower rate of clearance, but accounting for HCV genotype did not affect the estimates for SNPs or Aboriginal ancestry. Reduced power could also explain the lack of statistical significance of Aboriginal ancestry in the multivariate analysis. We also lacked the power to detect interactions between IFNL3 SNPs and sex which have been previously reported [55].

Our study population was very similar to the CCC overall so our results should be generalizable to co-infected individuals receiving care in Canada. However, although the CCC attempts to recruit from diverse populations including patients with various risk factors and who are marginalized, persons not accessing care may differ from those included in our analyses. Those not under care may be more unstable, active injection drug users and more likely to be Aboriginal. Furthermore, the CCC does not represent the full diversity of all Aboriginal people in Canada, but is most reflective of co-infected Aboriginals in the most populous Canadian regions (Ontario, British Columbia, Quebec and Alberta). Our results may not be fully generalizable to Aboriginal persons outside these regions.

Another potential limitation is that the date of HCV infection used as the origin was approximate in most instances. If the error of this estimation was differential by ethnic group, that is, greater in Aboriginals than whites, for example, then it could bias the effect estimate. However, when we modeled time at risk using age, which is known with better accuracy, or used different modeling strategies (discrete time survival analysis or conditional logistic in a nested case control study), we did not obtain different results (not shown). Since most individuals enrolled in the CCC many years after they were infected, we cannot know with certainty whether this was a first or repeat clearance. Nevertheless, our findings still address whether IFNL3 genotype is associated with spontaneous clearance in our study population.

In conclusion, HIV-HCV coinfected Aboriginals were more likely than whites to clear HCV infection and to carry the beneficial IFNL3 genotypes and alleles linked to increased HCV clearance. Future studies should explore the mechanisms behind enhanced clearance among Aboriginals, including functional studies of *IFNL3 and IFNL4* genes or any other host factors that might enhance the immune response to HCV infection. Understanding the underlying biology of HCV clearance will ultimately help in making treatment decisions for Aboriginals who have urgent clinical needs.

## 4. Materials and Methods

### 4.1. Source Population

The Canadian Co-infection Cohort Study (CCC, *n* = 1176), established in 2003, is an open prospective cohort of HIV-HCV co-infected individuals recruited from 18 centres across Canada, representing approximately 20% of the co-infected population under care [56]. For our study, we included data collected up until April 2013. To be included in the CCC, patients had to be over 16 years or older, give informed consent, be HIV infected (confirmed via ELISA with western blot), and have HCV infection or evidence of HCV exposure (HCV-antibody positive by ELISA with recombinant immunoblot assay II (RIBA II) or enzyme immunoassay (EIA) or if serologically false negative, HCV–RNA-positive). At visits every six months, socio-demographic, medical and behavioural information was collected using validated questionnaires and biological samples were obtained and stored. The study has been approved by research ethics boards at each of the participating institutions.

### 4.2. Study Population and Covariates

**Figure 3:**
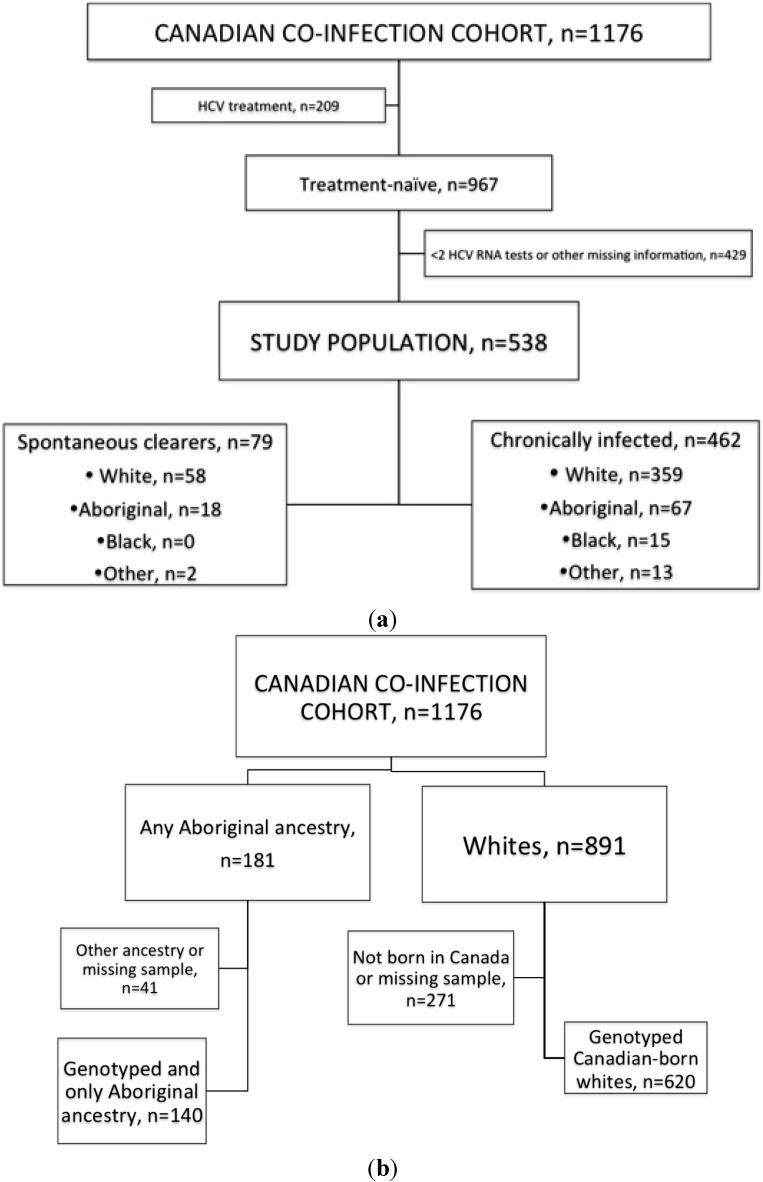
Study and source population: (**a**) Selection of study population for evaluating the association of IFNL3 genotypes and rates of spontaneous clearance; (**b**) Selection of study population for comparison of IFNL3 frequency distribution between Canadian Aborginals and Whites.

For the spontaneous clearance study, we included individuals who had never been treated for HCV and who had at least two HCV RNA tests available (*n* = 538). Visits after HCV treatment initiation were censored. A spontaneous clearance case was defined as an individual who was HCV-RNA negative on two consecutive PCR tests, at least six months apart (Figure 3a). HCV RNA levels were measured at most visits (COBAS AMPLICOR HCV Test, version 2.0, Roche Diagnostics, Hoffmann-La Roche Ltd., Laval, QC, Canada, lower limit of detection < 50 IU·mL^−1^).

To compare the genotype distribution of the three IFNL3 SNPs of interest between Canadian whites and Aboriginals, self-reported ethnicity was used. Participants self-identified as being of Caucasian, black, other (Asian or Hispanic Latino), or Aboriginal (First Nations, Metis, or Inuit) ethnicity. In the CCC, 15.6% reported some Aboriginal ancestry (*n* = 181), but analysis was restricted to those who did not report any other ancestry (*n* = 140). These results were compared to those from 620 genotyped Canadian-born whites (Figure 3b).

### 4.3. IFNL3 SNP Genotyping

Never thawed plasma and serum samples were processed and genotyped using a real-time PCR assay (Bay Area Genetic Lab). DNA was extracted using a modified Qiagen Mini Blood extraction protocol and the genotyping assay was developed for the LightCycler^®^ 480 (Roche Diagnostics, Laval, QC, Canada) to cover bi-allelic SNPs rs12979860, rs8099917, and rs8103142 separately. Oligos were designed in-house and synthesized by TibMolBiol. Each real-time assay consists of a primer set to amplify the specific gene region and a set of hydrolysis probes representing each allelic variant (common and rare allele). Probes are dual-labeled with a 5' reporter dye (6' FAM or HEX) and 3' quencher dye (BBQ). Results were analyzed using Endpoint Genotyping and Abs Quant/2nd derivative analysis software to determine the genotype at each SNP.

Samples with ambiguous or no result for an individual SNP were retested using more of the extracted DNA. For samples with ambiguous or no results for all the SNPs, more DNA was extracted and the SNP assays repeated using the newer extraction.

### 4.4. Statistical Analysis

The software PHASE v2.1 was used for haplotype inference and distribution [57,58]. Haploview [59] and Stata version 12 were used to determine allele frequency distributions, Hardy-Weinberg Equilibrium measures, and linkage disequilibrium (LD) measures in Aboriginals and Canadian-born whites. Pearson’s Chi-squared test was used to compare allelic and genotypic frequencies between the two subpopulations. The Stata command-hapipf- was used to test presence of LD in each group and also compare haplotypic frequencies between the two.

Due to the presence of censoring and left truncation, data were analyzed using Cox proportional hazards with adjustments for sex and ethnicity. The time axis was calendar time with the estimated date of HCV infection as the origin. Time in the analysis for each patient starts with cohort entry. This method of late entry was used to address the problem of left truncation since half the Cohort had been HCV infected for over 18 years at first visit. HCV duration was estimated based on date of HCV seroconversion, if known, or on the year of first injection drug use or blood product exposure. Interaction terms were tested between the IFNL3 genotype and sex as such interactions have previously been reported [55]. Due to the absence of HCV RNA in some available samples, HCV genotype information was missing in approximately 20% of the study population. Thus, HCV genotype was not included in the main multivariate analysis. In sensitivity analyses, Multiple Imputation by Chained Equations (MICE) was used to impute missing HCV genotypes [60]. Missingness for HCV genotype and other variables such as RNA tests, plasma samples, or IFNL3 genotype was assumed to be at random. Stata 12 was used for all analyses (StataCorp LP, College Station, TX, USA).

A dominant model was used in the association analyses between genotype and spontaneous clearance. Subjects with one or two copies of the variant allele were grouped and contrasted with the wild-type genotype. For all three SNPs the homozygous wild-type genotype is considered favourable. Therefore, for rs12979860, genotype CC was compared with the CT and TT genotypes, whereas for rs8099917 and rs8103142 the TT genotype was compared with the TG and GG genotypes or with the TC and CC genotypes, respectively.

For the haplotype analysis, the Stata command-haplologit- was used to test the effect of TCT, the haplotype with the favourable alleles at all the SNPs (T at rs8103142, C at rs12979860 and T at rs8099917), after adjusting for ethnicity.

## Appendix

The Canadian Co-infection cohort investigators (CTN222) are: Jeff Cohen, Windsor Regional Hospital Metropolitan Campus, Windsor, ON; Brian Conway, PENDER Downtown Infectious Diseases Clinic, Vancouver, BC; Curtis Cooper, The Ottawa Hospital—Research Institute, Ottawa ON; Pierre Côté, Clinique du Quartier Latin, Montréal, QC; Joseph Cox, MUHC IDTC- Montréal General Hospital, Montréal, QC; John Gill, Southern Alberta HIV Clinic, Calgary, AB; Shariq Haider, McMaster University Medical Centre—SIS Clinic, Hamilton, ON; Aida Sadr, Native BC Health Center, St-Paul’s Hospital, Vancouver, BC; Lynn Johnston, QEII Health Science Center for Clinical Research, Halifax, NS; Mark Hull, BC Centre for Excellence in HIV/AIDS, Vancouver, BC; Julio Montaner, St. Paul’s Hospital, Vancouver, BC; Erica Moodie, McGill University, Montreal, QC; Neora Pick, Oak Tree Clinic, Children’s and Women’s Health Centre of British Columbia, University of British Columbia, Vancouver, BC; Anita Rachlis, Sunnybrook & Women’s College Health Sciences Centre, Toronto, ON; Danielle Rouleau, Centre Hospitalier de l’Université de Montréal, Montréal, QC; Roger Sandre, Health Sciences North—The HAVEN/Hemophilia Program, Sudbury, ON; Joseph Mark Tyndall, Department of Medicine, Infectious Diseases Division, University of Ottawa, Ottawa ON; Marie-Louise Vachon, Centre Hospitalier Universitaire de Québec, Québec, QC; Steve Sanche, SHARE University of Saskatchewan, Saskatoon, SK; Stewart Skinner, Royal University Hospital & Westside Community Clinic, University of Saskatchewan, Saskatoon, SK; and David Wong, University Health Network, Toronto, ON.
